# Resilience and quality of life in patients who underwent mechanical ventilation due to COVID-19, one year after discharge: a cross-sectional study

**DOI:** 10.1186/s41687-024-00748-2

**Published:** 2024-07-12

**Authors:** David Rene Rodriguez Lima, Cristhian Rubio Ramos, Mateo Andrés Diaz Quiroz, Edith Elianna Rodríguez Aparicio, Leonardo Andrés Gómez Cortes, Laura Otálora González, Gilma Hernández-Herrera, Ángela María Pinzón Rondón, Ángela María Ruiz Sternberg

**Affiliations:** 1 Critical and Intensive Care Medicine, Hospital Universitario Mayor-Méderi, Bogotá, Colombia; 2 Grupo de Investigación Clínica, Escuela de Medicina y Ciencias de la Salud, Universidad del Rosario, Bogotá, Colombia; 3 Facultad de Medicina, Escuela de Medicina y Ciencias de la Salud, Universidad del Rosario, Bogotá, Colombia; 4 Doctorado Investigación Clínica, Escuela de Medicina y Ciencias de la Salud, Universidad del Rosario, Bogotá, Colombia

**Keywords:** Connor-Davidson Resilience Scale, Post-COVID-19 Functional Status Scale, EuroQoL 5D-3L, Quality of life, COVID-19, Mechanical ventilation, Resilience

## Abstract

**Background:**

Patients with COVID-19 often experience severe long-term sequelae. This study aimed to assess resilience and Quality of Life (QoL) of patients who underwent mechanical ventilation due to COVID-19, one year after discharge.

**Methods:**

This cross-sectional study enrolled patients who received mechanical ventilation for severe COVID-19 and were assessed one-year post-discharge. Participants completed a structured questionnaire via telephone comprising the Connor-Davidson Resilience Scale (CD-RISC) and the Post-COVID-19 Functional Status scale (PCFS). To establish the association between QoL and resilience, Spearman correlations were calculated between the PCFS and the CD-RISC. Linear regression models were adjusted to evaluate which factors were associated with QoL, with the total score of PCFS as the dependent variable.

**Results:**

A total of 225 patients were included in the analysis. The CD-RISC had a median score of 83 (IQR 74–91). The PCFS results showed that 61.3% (*n* = 138) of the patients were able to resume their daily activities without limitations. Among them, 37.3% (*n* = 84) were classified as Grade 0 and 24% (*n* = 54) as Grade 1. Mild and moderate functional limitations were found in 33.7% of the patients, with 24.8% (*n* = 56) classified as Grade 2 and 8.8% (*n* = 20) as Grade 3. Severe functional limitations (Grade 4) were observed in 4.8% (*n* = 11) of the patients. High CD-RISC scores were associated with lower levels of PCFS score (*p* < 0.001).

**Conclusions:**

In this cohort of critically ill patients who underwent mechanical ventilation due to COVID-19, 38% of patients experienced a significant decline in their QoL one year after hospital discharge. Finally, a high level of resilience was strongly associated with better QoL one year after discharge.

## Background

In March 2020, the SARS-CoV-2 pandemic, which causes Coronavirus-19 disease (COVID-19), was declared to have begun [1]. Between 10 and 20% of COVID-19 patients will require mechanical ventilation (MV) due to hypoxemic respiratory failure [2, 3].

The American Psychological Association dictionary defines resilience as the process and outcome of successful adaptation to a difficult life experience [4]. Resilience implies that a real threat must be present and that there needs to be an eventual adaptation or response that is considered adequate [5, 6].

During the COVID-19 pandemic, it has been demonstrated that higher levels of resilience, as measured by the Connor-Davidson Resilience Scale (CD-RISC) [7], have been associated with lower levels of anxiety, depression, and stress in COVID-19 survivors, particularly when the duration of COVID-19 was short [8].

The concept of quality of life (QoL) is complex and involves the satisfaction of multiple needs. While functionality in daily activities and the fulfillment of basic needs are significant pillars for this concept, they do not necessarily correlate with adequate QoL. The perception of being happy, fulfilled, and functional depends on many other factors, among which the ability of adaptation or resilience stands out [9]. According to Maslow, QoL is determined by the interaction among the individual, society, and the fulfillment of needs. It is established that individuals have different levels of needs, with some being more prioritized than others. Within this context, primary needs such as food, water, air, and security are initially addressed. Once these needs are met, other types of needs such as social acceptance, self-esteem, and self-actualization should be considered [10]. QoL is primarily incorporated into three branches of knowledge: economics, health, and social sciences. In each of these disciplines, the definition of QoL has been approached from a different perspective [11].

QoL is defined by the World Health Organization (WHO) as an individual’s perception of their overall well-being within the cultural context, the value system in which the individual lives, and regarding their goals, expectations, rules, and concerns [12].

Given the heterogeneity of COVID-19 and its impact on the population, simpler and reproducible tools have been developed to assess the impact of symptoms on patients’ QoL. A European multicenter group validated the Post-COVID-19 Functional Status Scale (PCFS) in 2020 for COVID-19 survivors, which could be evaluated from hospital discharge to six months of outpatient management, finding it useful to determine functional sequelae [13, 14]. De Jong et al. demonstrated the acceptance of the PCFS scale within the medical field, highlighting its simplicity and reproducibility as a tool for assessing QoL. Their findings provide evidence that the PCFS is a valuable and practical instrument that can be easily implemented in various healthcare settings [13, 15].

The EuroQol 5 dimensions 3 levels (EQ-5D-3L) questionnaire is an instrument where the individual assesses their health-related QoL, by rating their levels of severity in five dimensions: mobility, self-care, daily activities, pain/discomfort, and anxiety/depression [16, 17]. Several studies have evaluated its performance in COVID-19 survivors with reliable performance [18, 19].

The main objective of this study is to describe the resilience and QoL of critically ill patients who underwent MV due to COVID-19, one year after hospital discharge.

The secondary objectives include establishing the correlation between the PCFS and the EQ-5D-3L, as well as stablish which factors are associated with resilience and QoL in the same group of patients.

## Methods

### Design

A cross sectional study was conducted, and the present study received approval from the Institutional Ethics Committee (DVO005-1957- CV1534). Informed consent was obtained from each patient or their legal caregiver, which was given via telephone.

### Patients/population

Data were collected from patients who received MV support for respiratory failure secondary to critical illness due to COVID-19. The study included consecutively patients discharged alive from a high-complexity hospital located in the city of Bogotá, Colombia, from March 19, 2020, to April 30, 2021. The resilience and QoL questionnaires were applied from June 1, 2022, to December 31, 2022. Patients who passed away after discharge, those with whom telephone communication was not feasible, individuals with cognitive impairment preventing them from responding to the survey without obtaining information from a caregiver, and those who declined to participate in the study (as indicated by the patient and/or caregiver) were excluded from the final analysis.

### Intervention/measurement

Clinical characteristics, the presence, and severity of acute respiratory distress syndrome (ARDS), comorbidities quantified by Charlson Index, laboratory parameters obtained upon admission as well as discharge place and ambulatory treatment of the enrolled patients were assessed, this data was obtained from electronic register. Discharge place included the patient’s home, domiciliary hospitalization (this group of patients required completion of intravenous antibiotic medical therapy at home) or Long-Term Chronic Care Center (this group of patients needed chronic MV) and discharge treatment included home oxygen therapy, use of inhalers (short action beta agonist (SABA) and anticholinergic), respiratory and physical therapy and/or phonoaudiology.

Participants were administered a structured questionnaire that included three scales: CD-RISC, PCFS and EQ-5D-3L, all three administered via telephone application. Permission for the non-commercial use of the telephonic version of the EQ-5D-3L scale, was obtained from the EuroQoL Customer Portal (Registration No. 55,265).

### Statistical analysis

The general characteristics of the population and the results of the CD-RISC, PCFS and EQ-5D-3L were described. Continuous variables were presented as mean and standard deviation (SD) or median and interquartile range (IQR) depending on the data distribution. Categorical variables were described using absolute and relative frequencies. Initially, the items from each scale were described as categorical variables showing the total amount (n) and its percentage for each answer.

In the CD-RISC each one of the 25 dimensions was evaluated numerically and scored as follows: 0 for no alteration, 1 for mild alteration, 2 for moderate alteration, 3 for severe alteration, and 4 for critical alteration. The total CD-RISC score ranged from 0 (indicating no resilience) to 100 (indicating the best possible resilience).

The PCFS was reported using a grading system ranging from grade 0, indicating patients who were able to resume their daily activities without limitations, grade 1 negligible functional limitations, grade 2 slight functional limitations, grade 3 moderate functional limitations and grade 4, representing severe functional limitations.

The EQ-5D-3L scale evaluates five dimensions: mobility (Eq. 1), personal care (Eq. 2), daily activities (Eq. 3), pain/discomfort (Eq. 4), and anxiety/depression (Eq. 5), to evaluate the association between the EQ-5D-3L and the PCFS, each of these dimensions was considered as a numerical variable and they were scored as follows: Score 1: No alteration; Score 2: Moderate alteration; Score 3: Severe alteration. The total score was calculated by summing the scores from each of the five dimensions. The total score ranges from 5 (indicating optimal QoL with no alterations in any dimension) to 15 (indicating severely compromised QoL with severe alterations in all dimensions).

Polychoric correlations were individually made for EQ-5D-3L and CD-RISC. To confirm the validity of the PCFS a Spearman correlation between the PCFS and the EQ-5D-3L was made, based on the quantitative evaluation of the total score of the scales. Additionally, the association between resilience and QoL was made by Spearman correlation between the total scores of the CD-RISC, the PCFS and the EQ-5D-3L.

Furthermore, it was hypothesized that patients with higher resilience, as measured by the CD-RISC scale, would have better QoL. Spearman correlations were examined between the PCFS score and the total CD-RISC score.

To determine potential confounding factors that could influence the QoL of patients, the association between the Charlson Comorbidity Index, age, and PCFS was evaluated, this was made by the Kruskal-Wallis test considering each level of the PCFS as a categorical variable.

Considering that ambulatory treatment could also be a confounding factor, we assessed the association between discharge location, post-discharge treatments, and QoL. For this evaluation, PCFS was considered as a continuous variable, and the association was tested using the Kruskal-Wallis test for discharge location and the Mann-Whitney test for post-discharge treatments. This approach was adopted due to the non-normal distribution of PCFS.

Finally linear regression models were adjusted to evaluate which variables were associated with QoL and resilience.

## Results

**Fig. 1:**
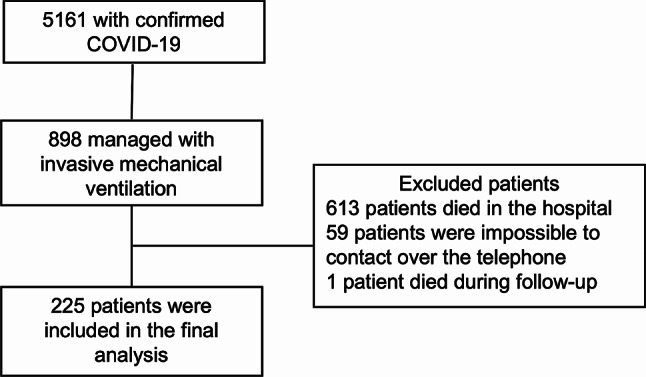
Patient recruitment flowchart. *COVID-19* Coronavirus-19 disease

There were 5161 patients diagnosed with COVID-19 between March 19, 2020, and April 20, 2021. Out of these, 898 required MV (17.3%) and 285 patients survived and were discharged (31.7%). In 59 cases, it was not possible to establish telephone communication, and one of these patients died, finally 225 patients were included in the final analysis according to the inclusion criteria (Fig. 1). The median age was 62 [53–70] years, with 60% being male. The most common comorbidity within the population was hypertension, present in 27.1% of patients. Regarding ARDS, 48 patients had mild, 122 moderate, and 49 severe pathology. Most patients (72.4%) were discharged at home, 22.6% required domiciliary hospitalization and only 3.1% required Long-Term Chronic Care Center. The most common ambulatory treatment was home oxygen therapy in 67.5% of patients, followed by inhalers in 36.8% of patients. Only 13.7% of patients received ambulatory physical therapy, 12.4% ambulatory respiratory therapy and 3.1% had follow up by phonoaudiology, as shown in Table 1.

**Table 1:** Clinical, demographic characteristics and inflammatory markers at hospital admission

*n* = 225	
General characteristics	
**Mean Age** [IQR]	62 [53–70]
**Sex** (n, %)	Male: 135 (60) Female: 90 (40)
**Mean Weight** (kg) [IQR]	75 [67–85]
**Education** (n, %)	Unfinished Elementary School: 38 (16.8)
	Finished Elementary School: 47 (20.8)
	Unfinished High School: 27 (12)
	Finished High School: 45 (20)
	Technical Degree: 37 (16.4)
	Undergraduate Degree: 22 (9.7)
	Postgraduate Studies: 9 (4)
**Comorbidities** (n, %)	Hypertension: 61 (27.1)
	Diabetes Mellitus: 42 (18.6)
	HIV– AIDS: 1 (0.44)
	COPD: 10 (4.44)
	Kidney disease: 8 (3.5)
	Malignancy: 0
	Rheumatic disease: 2 (0.89)
	Heart failure: 2 (0.89)
	Charlson Comorbidity Index (Mean [IQR]): 2 [1–3]
**Disease characteristics**	
**No ARDS** (n, %)	6 (2.6%)
**ARDS severity** (n, %)	Mild: 48 (21.3)
	Moderate: 122 (54.2)
	Severe: 49 (21.7)
**Tracheostomy requirement** (n, %)	39 (17.3)
**Mean inflammatory markers at ICU admission** [IQR]	Total white blood cell count (cells x 10^6^/L): 9870 [7070–14,060]
	Neutrophil/lymphocyte ratio: 9.2 [5.6–15.5]
	Hematocrit (%): 44.2 [41.2–47.2]
	Hemoglobin (g/dl): 14.8 [13.8–15.8]
	LDH U/L: 454 [371–597]
	D-dimer (ng/ml): 980 [612–1608.25]
	Procalcitonin (ng/ml): 0.37 [0.14–1.02]
	Ferritin (ng/ml): 1198 [880–1609]
**Discharge place** (n, %)	Home: 163 (72.4)
	Domiciliary hospitalization: 51 (22.6)
	Long-Term Chronic Care Center: 7 (3.1)
**Discharge treatment** (n, %)	Home oxygen therapy: 152 (67.5)
	Inhalers: 83 (36.8)
**Post-discharge therapy** (n, %)	Physical: 31 (13.7)
	Respiratory: 28 (12.4)
	Phonoaudiology: 7 (3.1)

*IQR* Interquartile range, *kg* kilogram, *HIV* Human immunodeficiency virus, *AIDS* Acquired immune deficiency syndrome, *COPD* Chronic Obstructive Pulmonary Disease, *ARDS* Acute Respiratory Distress Syndrome, *ICU* Intensive care unit, *L* liter, *g* grams, *dl* deciliter, *LDH* lactic dehydrogenase, *U* units, *ng* nanograms, *ml* milliliter

Table 2 summarizes the responses of the CD-RISC, scored on a continuous scale ranging from 0 to 100, with a median of 83 [74–91].

**Table 2:** CD-RISC questionnaire answers

Resilience					
Domains of the CD-RISC					
	0. Not true at all *n* (%)	1. Rarely true *n* (%)	2. Sometimes true *n* (%)	3. Often true *n* (%)	4. True nearly all of the time *n* (%)
1. Able to adapt to change	4 (1.7)	1 (0.44)	39 (17.3)	30 (13.3)	151 (67.1)
2. Close and secure relationships	6 (2.6)	1 (0.44)	18 (8)	13 (5.7)	187 (83.1)
3. Sometimes fate or God can help	0	3 (1.3)	11 (4.8)	7 (3.11)	204 (90.6)
4. Can deal with whatever comes	2 (0.88)	1 (0.44)	44 (19.5)	28 (12.4)	150 (66.6)
5. Past success gives confidence for new challenge	4 (1.7)	3 (1.3)	35 (15.5)	33 (14.6)	150 (66.6)
6. See the humorous side of things	1 (0.44)	11 (4.8)	88 (39.1)	44 (19.5)	81 (36)
7. Coping with stress strengthens	8 (3.5)	9 (4)	54 (24)	60 (26.6)	94 (41.7)
8. Tend to bounce back after illness or hardship	0	8 (3.5)	26 (11.5)	58 (25.7)	133 (59.1)
9. Things happen for a reason	10 (4.4)	28 (12.4)	38 (16.8)	33 (14.6)	116 (51.5)
10. Best effort no matter what	1 (0.44)	2 (0.88)	31 (13.7)	36 (16)	155 (68.8)
11. You can achieve your goals	1 (0.44)	2 (0.88)	31 (13.7)	36 (16)	155 (68.8)
12. When things look hopeless, I don’t give up	3 (1.3)	4 (1.7)	33 (14.6)	43 (19.1)	142 (63.1)
13. Know where to turn for help	1 (0.4)	0	23 (10.2)	25 (11.1)	176 (78.2)
14. Under pressure, focus and think clearly	14 (6.2)	12 (5.3)	55 (24.4)	36 (16)	108 (48)
15. Prefer to take the lead in problem solving	5 (2.2)	11 (4.8)	45 (20)	44 (19.5)	120 (53.3)
16. Not easily discouraged by failure	24 (10.6)	12 (5.3)	69 (30.6)	45 (20)	75 (33.)
17. Think of self as strong person	1 (0.44)	5 (2.2)	33 (14.6)	39 (17.3)	147 (65.3)
18. Make unpopular or difficult decisions	3 (1.33)	10 (4.44)	52 (23.1)	47 (20.8)	113 (50.2)
19. Can handle unpleasant feelings	2 (0.88)	17 (7.5)	57 (25.3)	56 (24.8)	93 (41.3)
20. Have to act on a hunch	77 (34.2)	48 (34.1)	43 (19.1)	9 (4)	18 (8)
21. Strong sense of purpose	1 (0.44)	1 (0.44)	20 (8.8)	43 (19.1)	160 (71.1)
22. In control of your life	11 (4.8)	11 (4.8)	42 (18.6)	45 (20)	116 (51.5)
23. I like challenges	15 (6.6)	11 (4.8)	44 (19.5)	43 (19.1)	112 (49.7)
24. You work to attain your goals	2 (0.88)	4 (1.7)	22 (9.7)	48 (21.3)	149 (66.2)
25. Pride in your achievements	0	1 (0.44)	19 (8.4)	38 (16.8)	167 (74.2)
*Total median [IQR]	**83 [74–91]**				

*IQR* Interquartile range, *CD-RISC* Connor-Davidson Resilience Scale, this scale has 25 dimensions with 5 levels each one

*Total median of the CD-RISC was calculated as a quantitative variable adding the value (levels 0–4) corresponding to each of the 25 dimensions, with the lowest value being 0 (severely comprise Resilience) and the highest value being 100 (optimal Resilience)

Table 3 presents the results of the two QoL scales. Firstly, the PCFS indicated that the most common response was at level 0, associated with optimal QoL, observed in 84 (37.3%) patients. Secondly, the EQ-5D-3L scale revealed that the most common response across the first dimension (mobility) was no mobility issues, regarding the second dimension which refers to personal care, 89.7% (*n* = 202) of patients reported no problems with it, among the third dimension, daily activities, 183 (81.3%) patients reported they had no issues with this aspect, the fourth dimension which evaluates pain or discomfort, 49.33% (*n* = 111) of patients reported it was moderately present, lastly in the fifth dimension, related to anxiety or depression, the level with more patients was no anxiety or depression reported by 130 (57.7%) patients.

**Table 3:** PCFS and EQ-5D-3L questionnaire answers

Quality of life	
PCFS (n, %)	Grade 0: 84 (37.3)
	Grade 1: 54 (24)
	Grade 2:56 (24.8)
	Grade 3: 20 (8.8)
	Grade 4: 11 (4.8)
	**Median [IQR]: 1 [0–2]
EQ-5D-3L (n, %)	**Mobility (EQ1))**
	1. No problems when walking: 171 (76)
	2. Some problems when walking: 51 (22)
	3. Stays in bed: 3 (1.3)
	**Personal care (EQ2))**
	1. No problems with personal care: 202 (89.7)
	2. Some problems with showering or dressing: 19 (8.4)
	3. Unable to shower or dress: 4 (1.7)
	**Daily activities (EQ3))**
	1. No problems performing daily activities: 183 (81.3)
	2. Some problems performing daily activities: 39 (17.3)
	3. Unable to perform daily activities: 3 (1.3)
	**Pain/discomfort (EQ4))**
	1. No pain or discomfort: 95 (42.22)
	2. Moderate pain or discomfort: 111 (49.33)
	3. Severe pain or discomfort: 19 (8.4)
	**Anxiety/depression (EQ5))**
	1. No anxiety or depression: 130 (57.7)
	2. Moderate anxiety and/or depression: 79 (35.3)
	3. Severe anxiety and/or depression: 16 (7.11)
	*Total median [IQR]: 6 [5–7]

*PCFS* Post-COVID-19 Functional Status Scale, this scale has only 1 dimension with 5 levels, with the lowest value being 0 (optimal Quality of Life) and the highest value being 4 (severely compromised Quality of Life), *EQ-5D-3L* EuroQol 5 dimensions 3 levels: This scale has 5 dimensions with 3 levels each one, *IQR* Interquartile range

*Total median of the EQ-5D-3L was calculated as a quantitative variable adding the value (levels 1, 2 or 3) corresponding to each of the 5 dimensions, with the lowest value being 5 (optimal Quality of Life) and the highest value being 15 (severely compromised Quality of Life)

**The median of the PCFS scale was calculated considering it as a quantitative variable

**Fig. 2:**
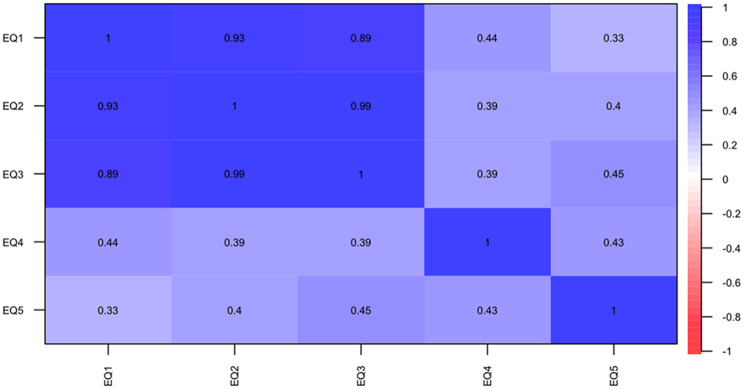
Polychoric correlations between each dimension of the CD-RISC. *CD-RISC* Connor-Davidson Resilience Scale (Dimensions 1–25)

Additionally, Fig. 2 shows polychoric correlations between each dimension of the CD-RISC, showing moderate correlations among different dimensions, except for dimension 20 (“Have to act on a hunch”), suggesting that this dimension may not be evaluating resilience in this population effectively.

**Fig. 3:**
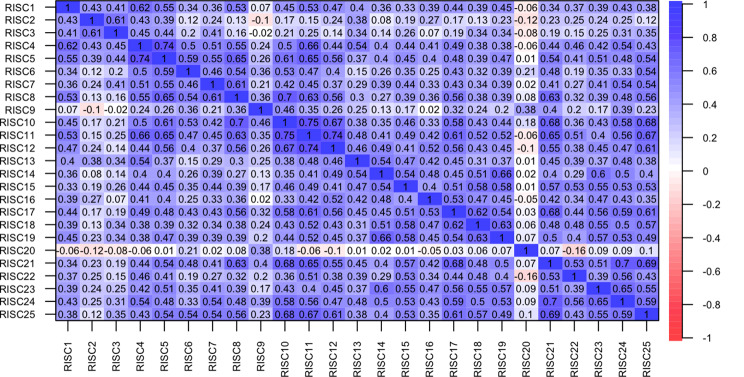
Polychoric correlations between each dimension of the EQ-5D-3L. *EQ* EuroQoL 5 dimensions 3 levels (1–5 dimensions)

Figure 3 illustrates polychoric correlations between each dimension of the EQ-5D-3L, indicating a high correlation among the first three dimensions (mobility, personal care, and daily activities) as expected due to their relation to motor abilities. A moderate correlation is observed between dimensions 4 and 5 (pain or discomfort and anxiety or depression) with the first three, suggesting they assess different aspects of QoL.

**Fig. 4:**
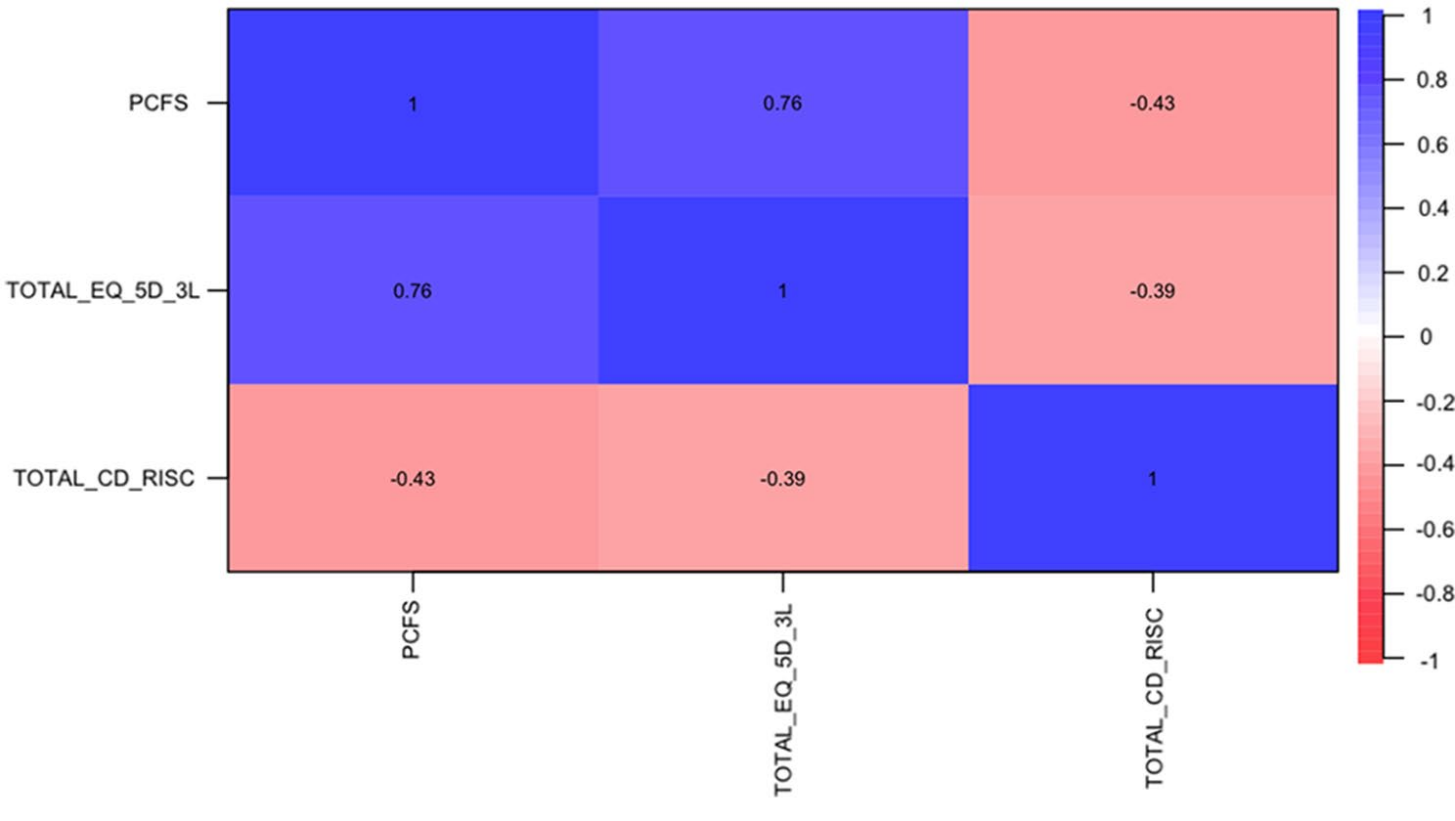
Spearman correlations between the total scores of CD-RISC, PCFS, EQ-5D-3L. *CD-RISC* Connor-Davidson Resilience Scale, *PCFS* Post-COVID-19 Functional Status Scale, *EQ-5D-3L* EuroQoL 5 dimensions 3 levels

Each dimension of the three scales evaluated was described firstly and then the total value of each scale was quantitatively analyzed for correlation with the other two scales. Spearman correlation coefficients revealed a high correlation between the three scales, specially it showed a strong correlation between the two QoL scales (PCFS and EQ-5D-3L) of 0.76 (*p* < 0.001). As shown in Fig. 4, it is indicated that lower scores on PCFS and EQ-5D-3L correspond to better QoL, and a higher score on CD-RISC indicates better resilience. Thus, negative but good correlations between the resilience scale the QoL scales were observed, with coefficients of −0.43 (*p* < 0.001) and −0.39 (*p* < 0.001) for PCFS and EQ-5D-3L, respectively.

**Fig. 5:**
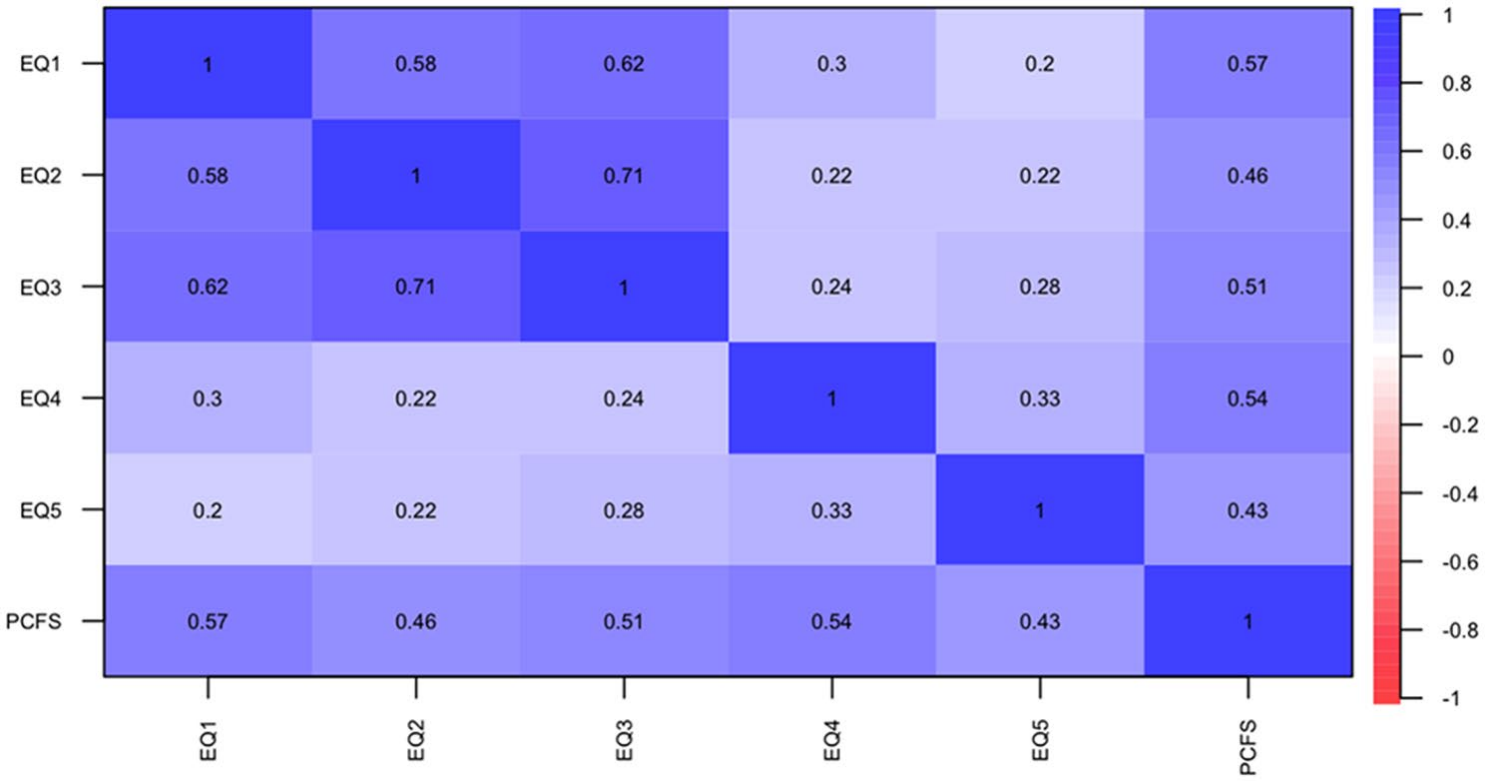
Spearman correlations between each dimension of the EQ-5D-3L and the PCFS. *EQ* EuroQoL 5 dimensions 3 levels (dimensions 1–5), *PCFS* Post-COVID-19 Functional Status Scale

**Fig. 6:**
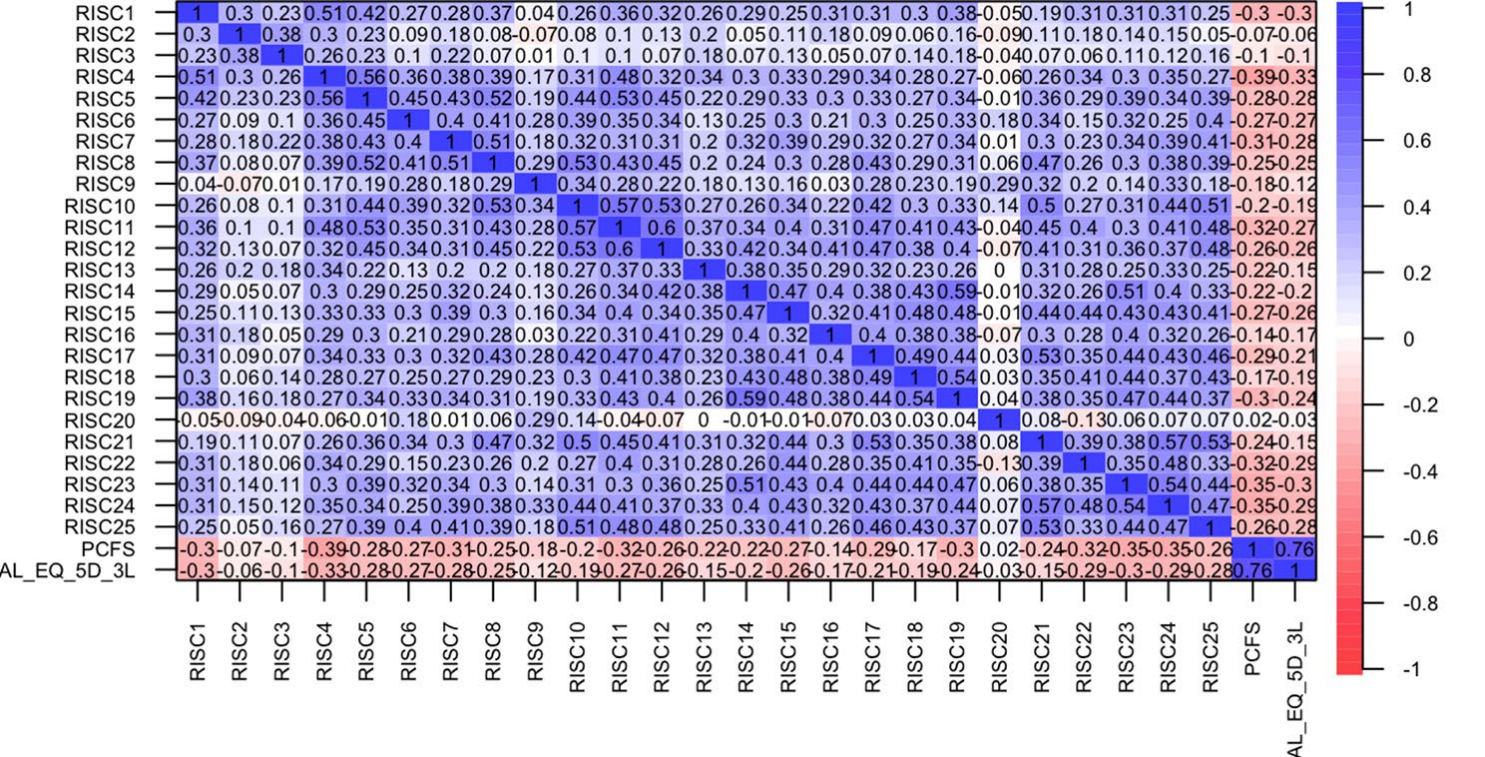
Spearman correlations between each dimension of the CD-RISC, the PCFS and the total score of the EQ-5D-3L. *CD-RISC* Connor-Davidson Resilience Scale (dimensions 1–25), *PCFS* Post-COVID-19 Functional Status Scale, *EQ-5D-3L* EuroQoL 5 dimensions 3 levels

Furthermore, Fig. 5 illustrates the correlation between PCFS and each dimension of EQ-5D-3L using Spearman correlation, indicating moderate correlations with all five dimensions. Correlations between each dimension of CD-RISC with the total score of the PCFS and the EQ-5D-3L are illustrated in Fig. 6, showing moderate negative correlations with each dimension of the two QoL scales.

**Fig. 7:**
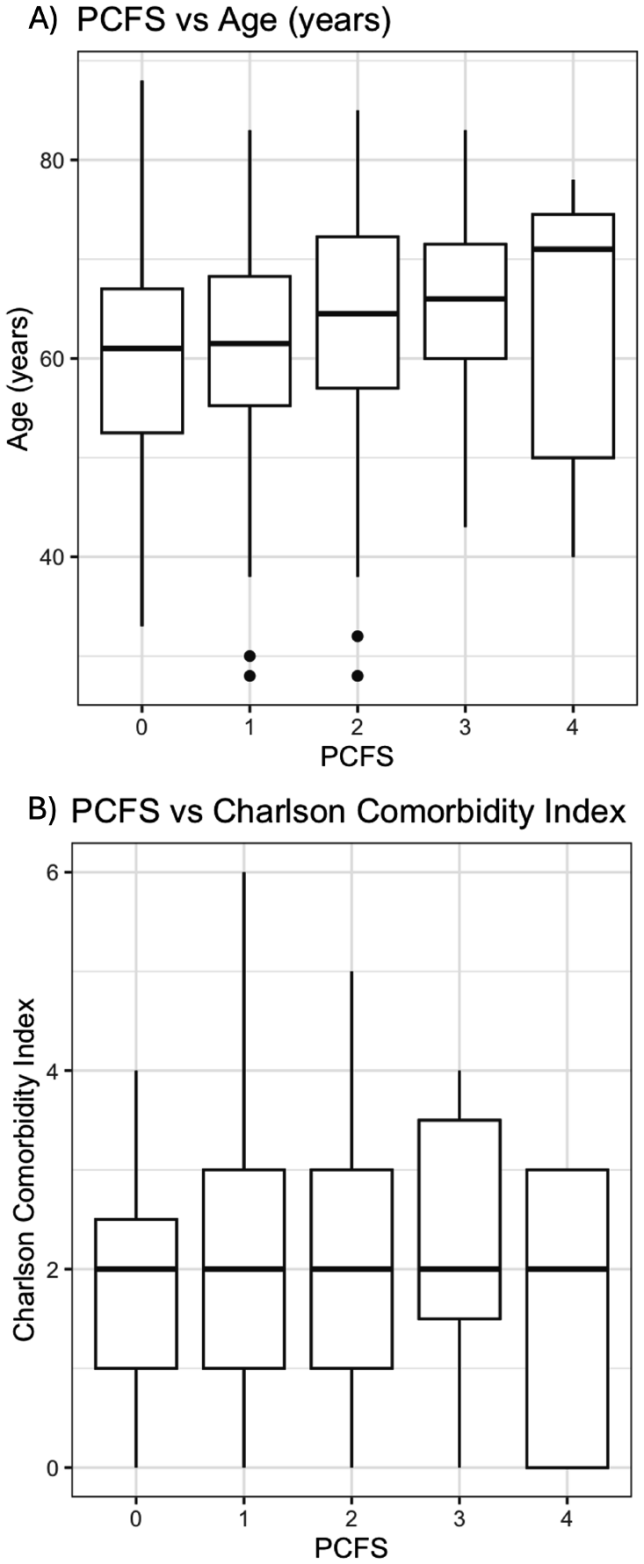
**A**) Association between PCFS and age. **B**) Association between PCFS and Charlson Comorbidity Index. *PCFS* Post-COVID-19 Functional Status Scale

No statistically significant association was found between patients’ age and their QoL measured by PCFS (*p* = 0.13) (Fig. 7A); nevertheless, there appears to be a trend indicating that with increasing age, QoL decreases. Additionally, no statistically significant association was found between Charlson Comorbidity Index and PCFS at discharge (*p* = 0.07) (Fig. 7B).

**Fig. 8:**
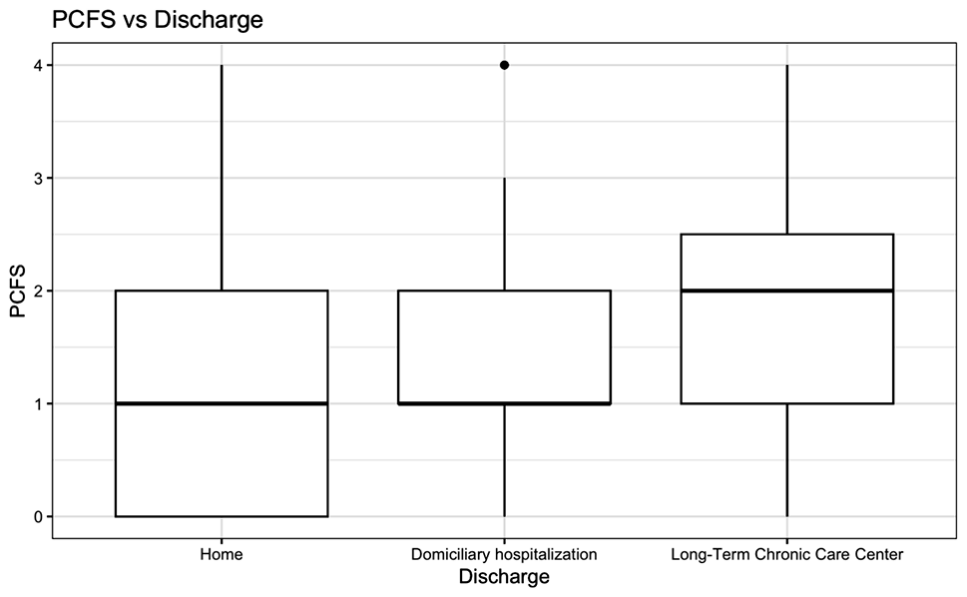
Association between PCFS and discharge place. *PCFS* Post-COVID-19 Functional Status Scale

**Fig. 9:**
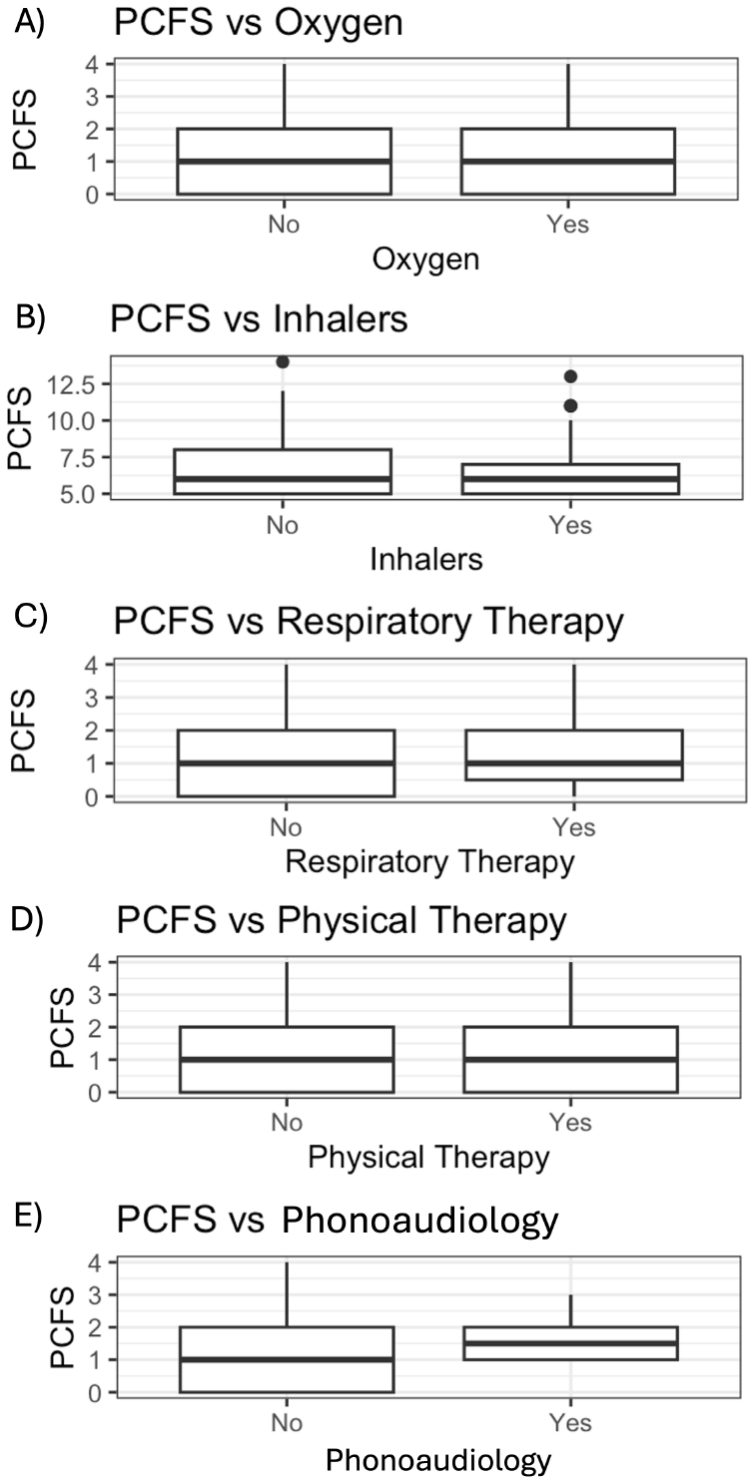
**A**) Association between PCFS and oxygen. **B**) Association between PCFS and Inhalers. **C**) Association between PCFS and Respiratory Therapy. **D**) Association between PCFS and Physical therapy. **E**) Association between PCFS and Phonoaudiology. PCFS: Post-COVID-19 Functional Status Scale

It was found that patients who required hospitalization in a Chronic Care facility had higher PCFS compared to those who were discharged home or to home hospitalization (*p* = 0.04) (Fig. 8). Conversely, no statistically significant association was found between the use of ambulatory oxygen (*p* = 0.13) or the use of inhalers at home (*p* = 0.74) and PCFS. Similarly, none of the evaluated therapies: respiratory (*p* = 0.49), physical (*p* = 0.58), and phonoaudiology (*p* = 0.45) showed statistically significant associations with PCFS (Fig. 9).

Finally, univariate, and multivariate models were conducted for CD-RISC (Table 4) and PCFS (Table 5) to determine variables affecting the total score of each scale. In the univariate analysis of CD-RISC as the dependent variable, significant variables included being men (*p* = 0.004), age (*p* = 0.008), weight (*p* = 0.02), and total PCFS score (*p* < 0.001), with only PCFS score (-4.23 [-5.57- -2.89]) and male gender (3.26 [0.06–6.47]) remaining significant in the multivariate analysis, suggesting that lower PCFS scores are associated with higher CD-RISC scores, indicating higher QoL, and male gender is associated with increased CD-RISC scores, indicating higher resilience. In the univariate analysis of PCFS, age (*p* = 0.03), Charlson index (*p* = 0.04), and total CD-RISC score (*p* < 0.001) were significant, with only the last one remaining significant in the multivariate analysis (-0.03 [-0.04- -0.02], p = < 0.001), suggesting that lower CD-RISC scores are associated with higher PCFS scores, indicating lower resilience and lower QoL, and vice versa.

**Table 4:** Univariate and multivariable linear regression analysis with CD-RISC as the dependent variable

Independent variable	Univariate model		Multivariable model	
	Unadjusted Beta Coefficient (95% CI)	*p*-value	Adjusted Beta Coefficient (95% CI)^a^	*p*-value
GenderMen	4.98 (1.56–8.41)	**0.004**	3.26 (0.06–6.47)	**0.04**
Age	-0.19 (-0.33- -0.04)	**0.008**	-0.24 (-0.49-0.005)	0.055
Charlson	-1.13 (-2.43-0.17)	0.08	1.54 (-0.70-3.78)	0.17
Weight	0.13 (0.01–0.25)	**0.02**	0.09 (-0.01-0.21)	0.09
No ARDS (reference)				
Mild ARDS	-4.14 (-15.26-6.97)	0.46	-4.61 (-15.09-5.85)	0.38
Moderate ARDS	-3.95 (-14.68-6.78)	0.46	-4.84 (-14.98-5.29)	0.34
Severe ARDS	-1.89 (-13.00-9.20)	0.73	-2.13 (-12.57-8.30)	0.68
PCFS	-4.46 (-5.79- -3.13)	**< 0.001**	-4.23 (-5.57- -2.89)	**< 0.001**

p-value for ANOVA < 0.001; R2 for the final multivariable model was 19.9%

Bold values indicate statistically significant p‐values (p < 0.05)

*CD-RISC* Connor-Davidson Resilience Scale, *CI* confidence interval, *ARDS* acute respiratory distress syndrome, *PCFS* Post-COVID-19 Functional Status Scale

**Table 5:** Univariate and multivariable linear regression analysis with a PCFS as the dependent variable

Independent variable	Univariate Model		Multivariable Model	
	Unadjusted Beta Coefficient (95% CI)	*p*-value	Adjusted Beta Coefficient (95% CI)	*p*-value
GenderMen	-0.29 (-0.61- 0.01)	0.06	-0.16 (-0.46-0.12)	0.26
Age	0.01 (0.00-0.02)	**0.03**	0.00 (-0.01-0.03)	0.55
Charlson	0.11 (0.00-0.23)	**0.04**	0.03 (-0.16-0.24)	0.71
Weight	0.00 (-0.007-0.01)	0.58	0.01 (-0.0001-0.02)	0.052
ARDS (reference)				
Mild ARDS	0.54 (-0.46-1.54)	0.28	0.36 (-0.60-1.32)	0.45
Moderate ARDS	0.48 (-0.48-1.45)	0.32	0.26 (-0.66-1.20)	0.57
Severe ARDS	0.70 (-0.30-1.70)	0.17	0.52 (-0.43-1.48)	0.28
TOTAL_CD_RISC	-0.03 (-0.04- -0.02)	**< 0.001**	-0.03 (-0.04- -0.02)	**< 0.001**

p-value for ANOVA < 0.001; R2 for the final multivariable model was 19.9%

Bold values indicate statistically significant p‐values (p < 0.05)

*PCFS* Post-COVID-19 Functional Status Scale, *CI* confidence interval, *ARDS* Acute respiratory, *CD-RISC* Connor-Davidson Resilience Scale

## Discussion

Post-pandemic management of severe COVID-19 survivors requires a multidisciplinary approach, including monitoring pulmonary and cardiovascular function, assessing inflammation, and evaluating QoL [20–22].

Assessing the QoL in post-COVID-19 patients who underwent MV is currently a topic of great interest in public health [23]. In a study conducted by Zangrillo et al., a follow-up was conducted on 56 patients who received MV in a COVID-19 intensive care unit (ICU) and were subsequently discharged from the hospital for one year. The study utilized multiple scales to assess various dimensions of QoL. The findings revealed a low mortality rate at one year, with the majority of patients demonstrating satisfactory performance in their daily activities in both physical and psychological dimensions. [24]. However, the use of multiple scales in the clinical setting may not be practical.

This study includes one of the largest patient cohorts with long-term resilience and QoL follow-up after one year of ICU discharge following MV for severe COVID-19. The results reveal a severe disability rate of 4.8%. Moreover, it was observed that up to 38% of the patients experienced some degree of disability, which aligns with previous reports in the literature [25–29].

Differing from expectations, neither age nor comorbidities were associated with changes in QoL at discharge. However, this could be explained by the fact that this cohort was very homogeneous, as all patients survived and had severe COVID-19 requiring mechanical ventilation. It is likely that older patients with more comorbidities had died during hospitalization. Consequently, those who survived were relatively younger and had fewer comorbidities.

Neither home oxygen therapy, nor the use of ambulatory inhalers, nor the implementation of different ambulatory rehabilitation therapy strategies turned out to be associated with changes in QoL. Nevertheless, it is noteworthy that a low number of patients underwent ambulatory therapy and phonoaudiology. This could be explained by the limitation of medical ambulatory resources during the first year of the pandemic.

As expected an association between high PCFS and discharge to Long-Term Chronic Care Centers was found, as more than an ambulatory care strategy it indicates that in this group of patients, disability at discharge was very high [30].

In contrast, patients who were discharged to their home showed lower PCFS scores than those with domiciliary hospitalization or discharged to Long-Term Chronic Care Centers, this could highlight the importance of going back to their natural environment in the perception of QoL further than in their disability: as explained by Masglow’s theory [10], QoL is determined by the interaction of the individual with society and the fulfillment of their needs.

Resilience is a key factor in post-critical illness QoL. [31, 32]. Only a limited number of studies have investigated resilience in COVID-19 patients who underwent MV. In New York State, USA, during the pandemic, a psychological support hotline was established to assist 251 patients via telephone between March and May 2021, resulting in improved resilience skills. [33]. The intervention had a positive impact on post-traumatic stress scales, indicating the effectiveness of support strategies, fostering connections with others, and enhancing problem-solving abilities even in challenging circumstances. Higher levels of resilience, as assessed by the 10-item CD-RISC, have been linked to lower levels of anxiety, depression, and stress in patients who have survived COVID-19, particularly when the duration of illness was relatively short. These findings indicate the important role of resilience in promoting psychological well-being and coping with the challenges associated with COVID-19 recovery [7, 8].

In the post-COVID-19 population, the EQ-5D-3L scale has been validated and demonstrates acceptable performance as a measure of QoL [25–29]. Despite the validation and acceptable performance of the EQ-5D-3L scale as a measure of QoL in the post-COVID-19 population, there are still challenges associated with the scale. One notable challenge is the modification from 3 to 5 dimensions, which may impact its diagnostic and prognostic capacity. It is important to consider these limitations when interpreting the results obtained using the EQ-5D-3L scale in the context of post-COVID-19 QoL assessments. [34–37]. Furthermore, it is worth noting that the EQ-5D-3L scale may require extensive questioning, which can be a potential limitation. In contrast, the PCFS, validated in 2020, offers a user-friendly and easily applicable tool for measuring QoL. The PCFS provides a more streamlined and convenient approach to assessing QoL in post-COVID-19 patients [14], and it has been widely used by many authors [13, 38]. De Jong et al. demonstrated the acceptance of the PCFS scale within the medical field, highlighting its simplicity and reproducibility as a tool for assessing QoL. Their findings provide evidence that the PCFS is a valuable and practical instrument that can be easily implemented in various healthcare settings [13].

Indeed, this study represents the only available research that assesses the performance of the PCFS scale in comparison to the EQ-5D-3L across its various domains. The findings indicate a strong correlation between the PCFS and EQ-5D-3L in domains such as mobility, self-care, and daily activities, demonstrating very good agreement. The correlation was also good for the presence of pain or discomfort and acceptable for the presence of depression and anxiety. Furthermore, when the entire EQ-5D-3L scale was considered in conjunction with the PCFS, the correlation was even higher than that observed for each individual dimension. This indicates that the PCFS is a dependable tool for evaluating the QoL in patients who have recovered from COVID-19. Therefore, in this paper the PCFS was used as the quantitative value representing QoL.

In this study, resilience was assessed in COVID-19 patients who underwent MV, and the findings demonstrated high levels of resilience in this population. Moreover, the study revealed a significant association between higher levels of resilience and lower PCFS scores, indicating better functional status and QoL in the post-COVID-19 period. These results are consistent with previous studies conducted in various countries (China, USA, and Mexico) and among individuals with diverse socioeconomic backgrounds, using different validated measurement scales in non-pandemic settings. [8, 33, 39].

This study has limitations due to its single-center nature, which restricts the generalizability of the findings. Nevertheless, it is the largest cohort study to date that reports on QoL and resilience in patients managed with MV during the pandemic; taking into account that there have been published other resilience and QoL papers but with other type of patients such as breast cancer [40], colon cancer [41] and epilepsy [42] as well as those regarding the caregivers instead of the patient itself [43].

The questionnaires were administered by trained medical personnel, reducing measurement bias. However, not all measurements were repeated for each patient, limiting the evaluation of intra and inter-observer reliability.

Finally, the study demonstrates that QoL is negatively impacted in approximately 38% of patients who experienced severe COVID-19. The PCFS score proves to have a negative association with the CD-RISC score, which shows that at lower values of PCFS (better QoL), higher values of CD-RISC (better resilience), therefore resilience is associated with improved QoL.

## Conclusion

In this cohort of critically ill patients who underwent MV due to COVID-19, 38% of patients experienced a noteworthy decline in their QoL one year after hospital discharge, those with higher resilience levels had better QoL one year after discharge. Additionally, the PCFS showed a strong correlation with the EQ-5D-3L, indicating its utility as a simple and effective tool for evaluating QoL in this population.

## Data Availability

Dataset is available at DOI: 10.5281/zenodo.10734227.

## Abbreviations

COVID-19: Coronavirus-19 disease
Quality of Life: QoL
MV: Mechanical Ventilation
CD-RISC: Connor-Davidson Resilience Scale
PCFS: Post-COVID-19 functional Status Scale
EQ-5D-3L: EuroQoL 5 dimensions 3 levels
WHO: World Health organization
ARDS: Acute Respiratory Distress Syndrome
SD: Standard deviation
IQR: Interquartile range
ICU: Intensive care unit
USA: United States of America
HIV: Human immunodeficiency virus
AIDS: Acquired immune deficiency syndrome
g: Grams
dl: Deciliter
LDH: Lactic dehydrogenase
ng: Nanograms
ml: Milliliter
